# In-Fiber Mach-Zehnder Interferometer Based on Three-Core Fiber for Measurement of Directional Bending

**DOI:** 10.3390/s19010205

**Published:** 2019-01-08

**Authors:** Lei Ding, Yu Li, Cai Zhou, Min Hu, Yuli Xiong, Zhongliang Zeng

**Affiliations:** Hubei Province Engineering Research Center for Intelligent Micro-Nano Medical Equipment and Key Technologies, School of Electrical and Electronics Engineering, Wuhan Textile University, Wuhan 300020, China; szhou@wtu.edu.cn (C.Z.); hm@wtu.edu.cn (M.H.); ylxiong@wtu.edu.cn (Y.X.); zzl@wtu.edu.cn (Z.Z.)

**Keywords:** three-core fiber, Mach-Zehnder interferometer, bending sensitivities, bending direction, fiber optic sensors

## Abstract

A highly sensitive directional bending sensor based on a three-core fiber (TCF) Mach-Zehnder interferometer (MZI) is presented in this study. This MZI-based bending sensor was fabricated by fusion-splicing a section of TCF between two single-mode fibers (SMF) with core-offset. Due to the location of the core in the TCF, a bend applied to the TCF-based MZI led to an elongation or shortening of the core, which makes the sensor suitable for directional bending measurement. To analyze the bending characteristics, two types of TCF-based sensors, with the fusion-spliced core located at different positions between the SMFs, were investigated. A swept source was employed in the measurement technique. The experimental results showed that, for the two types of sensors in this setup, the bending sensitivities of the two sensors were 15.36 nm/m^−1^ and 3.11 nm/m^−1^ at the bending direction of 0°, and −20.48 nm/m^−1^ and −5.29 nm/m^−1^ at the bending direction of 180°. The temperature sensitivities of the two sensors were 0.043 nm/°C and 0.041 nm/°C, respectively. The proposed sensors are compact, versatile, inexpensive to fabricate, and are expected to have potential applications in biomedical sensing.

## 1. Introduction

Optical fiber interferometer sensors are important in the fields of robotics, structural monitoring, and the automotive and aerospace industries, because of their compact size, integrated structure, and low loss [1,2]. Many types of sensors have been introduced, such as tilted fiber Bragg gratings (FBG) [3], long period fiber gratings (LPFG) [4,5], gratings written in specific fibers [6,7], and in-fiber interferometers [8,9]. These configurations possess many advantages and can accurately measure bending direction [10].

Generally, bending sensors are fabricated using FBG configurations. Specifically, some researchers detected bending direction by fabricating general FBGs or LPFGs in specially-designed fibers. These include eccentric core fibers (ECF) [11,12], multi-core fibers [13], holey fibers [14], multi-mode fibers (MMF) [15] or photonic crystal fibers (PCF) [16], D-shaped cladding fibers [17] and general fibers that have asymmetrical refractive index gratings inscribed into them with a laser [18,19,20,21,22]. However, the FBG- and LPFG-based directional bending sensors are complicated and expensive to fabricate. In addition, both these sensor types have low bending sensitivities. Alternatively, in-fiber interferometers can be used for directional bending detection by offsetting the cores, such as in single mode fibers (SMF) [23,24,25,26] and in PCF [27]. For these in-fiber interferometers, bending direction is detected by using asymmetrical structures, such as asymmetrical tapers, micro notches, or lateral-offset fusion splice junctions in the SMFs, and a peanut-like section and an abrupt taper in PCF-based Mach-Zehnder interferometers (MZI) [27]. These bending sensors can be easily fabricated and are highly sensitive, and are thus suitable for the fields of gait pattern detection, such as measuring the change in joint angles.

This paper proposes an in-fiber MZI based on three-core fiber (TCF) for directional bending recognition. The structure consists of a TCF with its ends spliced separately with single-mode fibers (SMFs). To analyze the characteristics of the MZI, two types of sensors with different core locations in the TCF were fusion-spliced together with SMFs. A swept source was used in the measurement technique. The experiments simulated different bending environments to demonstrate that TCF-based MZIs have regular characteristics. This configuration produced higher bending sensitivities with no insensitive region, and is simple to fabricate in contrast with the FBGs- and LPFG-based structures. Compared with the photonic crystal splice sensor, which has better sensitivity reported in Ming Deng et al. [28], the configuration is less expensive to fabricate. The sensor also has attractive features such as compact size and ease of demodulation. This design’s low fabrication cost and versatility are attractive for applications in biomedical fields where monitoring of bends is required, such as for sensing the trajectories of joint angles.

## 2. Experimental Procedures and Methods

### 2.1. Sensor Fabrication and Principles

**Figure 1:**
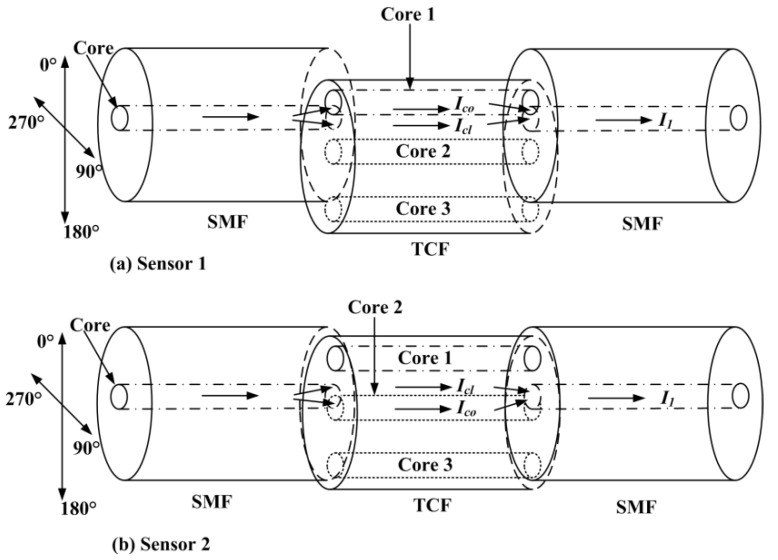
Schematic structure of the TCF-based Mach-Zehnder Interferometer. SMF: Single mode fiber; TCF: Three core fiber; *I_co_*: The light intensity of the core mode; *I_cl_*: The light intensity of the dominant cladding mode; and *I*_1_: The intensity of the interference signal.

**Figure 2:**
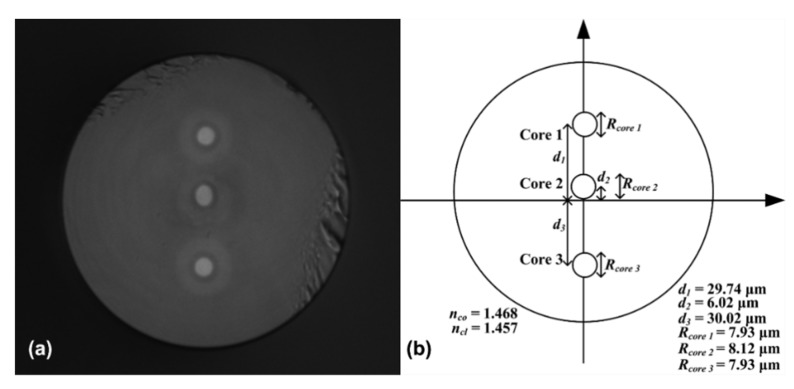
(**a**) Microscope image of the cross-sections of the TCF. (**b**) Schematic of the distribution of the three cores. *d*_1_: The distance between Core 1 and the neutral plane; *d*_2_: The distance between Core 2 and the neutral plane; *d*_3_: The distance between Core 3 and the neutral plane; *R_core_*_1_: The diameter of Core 1; *R_core_*_2_: The diameter of Core 2; *R_core_*_3_: The diameter of Core 3; *n_co_*: The refractive indexes of the core mode; *n_cl_*: The refractive indexes of the cladding mode.

The structures of the TCF-based sensors (sensor 1 and sensor 2) are shown in Figure 1a,b, respectively, and both included two parts: SMFs and TCF. An image of the designed TCF and a schematic of the distribution of the three cores are shown in Figure 2a,b, respectively. The cores in the TCF (*d*_1_, *d*_2_, and *d*_3_) were 29.74 µm, 6.02 µm, and 30.02 µm away from the central axis in the TCF, respectively. The TCF included three cores, and the diameter of the cores (*R_core_*_1_, *R_core_*_2_, and *R_core_*_3_) were 7.93 µm, 8.12 µm, and 7.93 µm, respectively. The refractive indexes (RI) of the core mode (*n_co_*) and cladding mode (*n_cl_*) were 1.468 and 1.457, respectively. The TCF was fusion-spliced between two standard SMFs with lateral core-offset by using a fusion splicer (Fujikura, 80S, Japan). 

The sensing element was fabricated using the following four steps: one end of the face of the SMF and TCF were connected to the swept source and the optical power meter (8153A, Agilent, Santa Clara, CA, USA). Next, we spliced the other end face of the SMF and TCF in the fiber fusion splicer. During the process, the relative positions of the SMF and TCF in the X-axis and Y-axis were adjusted in “Manual” mode. When the monitoring value of the optical power meter was at the maximum, it indicated that the cores of the SMF and TCF were aligned on-axis. Third, we recorded the maximum value of the optical power meter and continued to adjust its relative position. We started splicing when the value decayed to 50%, because this indicated that the SMF and TCF were spliced with about a 4 µm core-offset. Finally, after cutting the TCF, the cut end face was spliced to one end face of the second SMF. The other end face of the SMF was connected to the optical spectrum analyzer (OSA). When adjusting their relative position, the intensity of the interference signals underwent a periodic change which gradually increased, decreased, then gradually increased, and then decreased. When the intensity of the interference signals was the highest, the other end face of the TCF was fusion spliced to the SMF with a small lateral core-offset (about 4 µm). Notably, while splicing the TCF to the SMF, the reflected interference signal was detected in real time to determine the contrast ratio of the fringe with an OSA. The fiber fusion splicer was operated in “Manual” mode, the fiber type was selected as “Single-Single” (SS), the arc discharge current was set to standard −25 bit, and the arc duration was 350 ms. 

The device operates on the following principles. When the input light of the swept source reaches the first junction between the SMF and TCF, cladding modes of the TCF are excited. At the second offset junction, the core and the cladding modes of the TCF are re-coupled back to the core of the second SMF, which results in an interference fringe because of the phase difference. In addition, more high-order cladding modes are stimulated in the structure.

**Figure 3:**
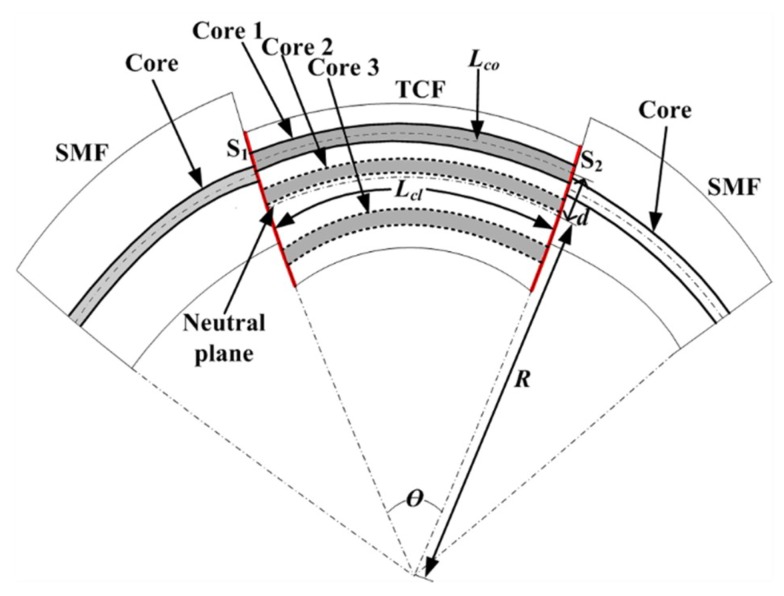
Schematic view of a curved TCF-based Mach-Zehnder Interferometer (MZI). SMF: Tingle mode fiber; TCF: Three core fiber; S_1_: The SMF-TCF interface; S_2_: The TCF-SMF interface; *d*: The distance between the eccentric core and the neutral plane; *R*: The radius of curvature.

The schematic view of bending is shown in Figure 3. The angle between S_1_ and S_2_ (i.e., the SMF-TCF and TCF-SMF interfaces) is:

(1) $$\theta \approx \frac{L_{cl}}{R}=\frac{L_{co}}{R+d}$$

where *R* is the radius of curvature, *d* is the distance between the eccentric core and the neutral plane of the TCF during bending, and *L_cl_* and *L_co_* are the length of the cladding (same as the length of the TCF) and the core of the TCF, respectively. Equation (1) can be rewritten as:

(2) $$L_{co}=\left(1+\frac{d}{R}\right)\cdot L_{cl}$$

Then, Equation (2) can be simplified as:

(3) $$L_{co}=\left(1+d\cdot C\right)\cdot L_{cl}$$

where *C* is the bending curvature. The phase difference *γ* between the core and the cladding mode after transmission along the TCF, which can be expressed as [29]:

(4) $$\gamma =\frac{2\pi }{\lambda }\left(n_{co}L_{co}-n_{cl,i}L_{cl}\right)$$

where *n_co_* and *n_cl,i_* are the effective RIs of the core mode and the *i*th-order cladding mode, respectively, and *λ* is the wavelength of the light source. When phase difference satisfies γ=(2k+1)⋅π, where *k* is the order of the MZI, the wavelength of the resonant dip can be computed as:

(5) $$\lambda_{d}=\frac{2(n_{co}L_{co}-n_{cl,i}L_{cl})}{2k+1}$$

When a bending is applied on the TCF-based MZI, *n_co_* and *L_co_* of the core mode will suffer a distinct change due to the bend-induced strain on the eccentric core. However, *n_cl,i_* and *L_cl_* of the cladding mode will change less than those of the core mode. Specifically, for the bending of 0° direction in the experiments, the core of the TCF was stretched and the optical path of the core mode increased more than that of the cladding mode. As a result, the optical path difference between the core mode and cladding modes ($n_{co}L_{co}-n_{cl,i}L_{cl}$) increased, leading to an increase in the wavelength of the interference minima. For the bending of 180° direction in the experiments, the core of the TCF was contracted and the optical path difference between the core and cladding modes ($n_{co}L_{co}-n_{cl,i}L_{cl}$) decreased, leading to a decrease in the wavelength of the interference minima. At the 90° and 270° direction, the optical path difference between the core mode and cladding mode was constant because the cores were in the neutral plane. 

According to Equations (3) and (5), the wavelength difference variation is related to the optical path difference between the core mode and cladding modes, the distance between the eccentric core and the neutral plane, and the curvature difference. As a result, this TCF-based MZI shows potential for application in directional bending sensing.

### 2.2. Experimental Setup

**Figure 4:**
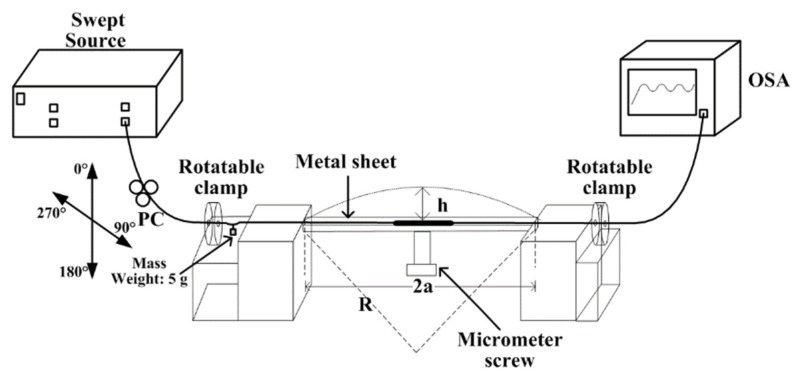
Experimental setup for directional bending measurement under different directions and curvatures. PC: Polarization controller; OSA: Optical spectrum analyzer.

As shown in Figure 4, an experimental setup was established to analyze the bending characteristics of the TCF-based MZI sensors. A laboratory-built swept source was used as the light source, which mainly included a semiconductor optical amplifier (IPSAD1304, InPhenix, Livermore, CA, USA), a scanning Fabry-Perot filter (FFP-TF2, Micron Optics, USA), a function generator (AFG1000, Tektronix, Atlanta, GA, USA), polarization controllers (PC), fiber couplers, and isolators. The scanning range of the swept source was 1285 to 1348 nm, with an instantaneous line-width of 0.02 nm. The output power was 8.4 mW at a sweeping rate of 2.5 kHz. Since the instantaneous linewidth of 0.02 nm produced a coherence length of approximately 37.18 mm, the total effective length difference between the core traverse and the cladding traverse was well below 37.18 mm.

The output SMF of the swept source was connected to a PC, and the PC was connected to the rotatable clamps. Two fiber holders were employed to hold the fiber sensor, and a steel sheet (100 mm × 20 mm × 1 mm) was placed on top of them to cover the sensing element. At both sides of the two fiber holders, the rotatable clamps were fixed to adjust the bending direction. The rotation range was 0–360° in steps of 5°. A 5-g mass was hung in the middle of the rotatable clamp and the holder. As a result, the sensor could be kept in close contact with the steel sheet when bending was applied. A precise micrometer screw was used to press the steel sheet and apply the bending to the sensing head. The transmission spectrum from the pigtails of the sensor was measured by the OSA (YOKOGAWA AQ6370C). 

### 2.3. Bending Experiments

In order to analyze the polarization effects during the experiments, the response of the sensor under different polarizations was investigated. The process was carried out following two steps: firstly, placing the sensor horizontally and setting the bending direction of the sensor at 0°, using the PC to adjust the polarization in steps of 45° and to monitor the OSA response. Secondly, we set the bending direction and curvatures at 180° and 0.5 m^−1^, respectively, using the PC to adjust the polarization to steps of 45° and monitor the OSA response. We clearly observed from the above experiments that the spectrum did not shift and the spectral response of the monitoring was not affected when adjusting the polarization. Thus, the polarization was parallel to the bending plane, which ensured consistency of the experiment.

The sensing characteristics of the TCF-based MZI sensors were investigated under different directions and curvatures. Specifically, two types of the sensors with different core locations in TCFs were fabricated. Sensor 1 was fabricated with an eccentric core (Core 1) in the TCF fusion-spliced between SMFs, with a core-offset of 4 µm. Sensor 2 was fabricated with an eccentric core (Core 2) in the TCF fusion-spliced between SMFs, with a core-offset of 4 µm. The lengths of the TCF of the sensors were 35.1 mm (sensor 1) and 35.5 mm (sensor 2). For each TCF-based sensor, four typical bending directions (0°, 90°, 180°, and 270°) were measured during the experiments. The direction of 270° made the eccentric-core occur in the neutral plane, as did 90°. Then, for each direction, the curvatures ranged from 0 to 1 m^−1^.

## 3. Results and Discussion

### 3.1. Transmission Spectra of the Sensors

**Figure 5:**
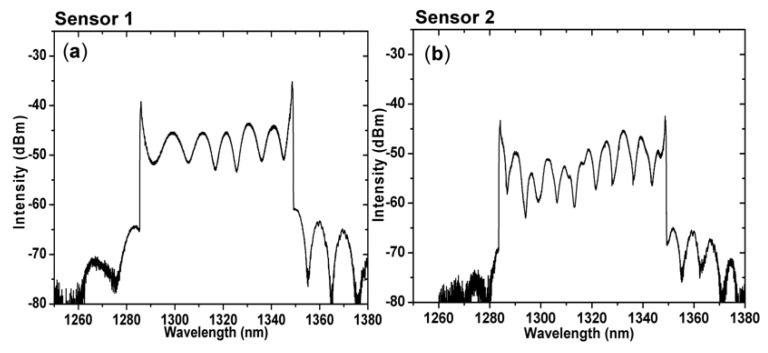
Transmission spectrum of the TCF-based MZI (**a**) sensor 1 and (**b**) sensor 2.

Figure 5a,b shows the transmission spectrum of the TCF-based MZI sensors using a swept source at a sweeping rate of 2.5 kHz at room temperature (25 °C). The average wavelength separations of sensors 1 and 2 were 12.5 nm and 10.5 nm, respectively. The contrast ratios were 10 dB and 8 dB, respectively. Higher-order modes were excited to construct the MZI, which means the transmission spectrum of the sensors had a higher frequency component. The excited cladding modes of the sensors were not exactly the same. Each interference spectrum had multiple cladding modes, therefore, an uneven interference spectrum could have been caused by involving multiple cladding modes. Owing to the different lengths of the two sensors, the difference in the optical path lengths were different. As a result, the free spectrum ranges (FSRs) of the two sensors were not identical.

### 3.2. Wavelength Shift of the TCF-Based Sensor in Bending Experiments

**Figure 6:**
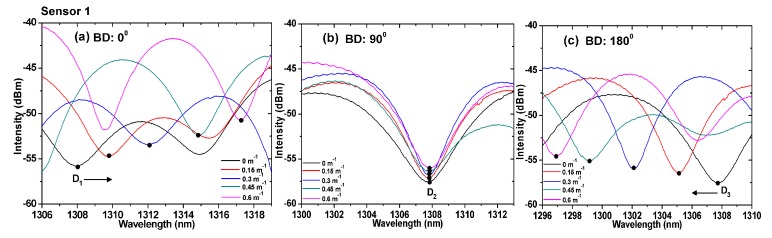
Spectra of TCF-based sensor 1 under different curvatures for bending directions of (**a**) 0°, (**b**) 90°, and (**c**) 180°. BD: Bending direction.

**Figure 7:**
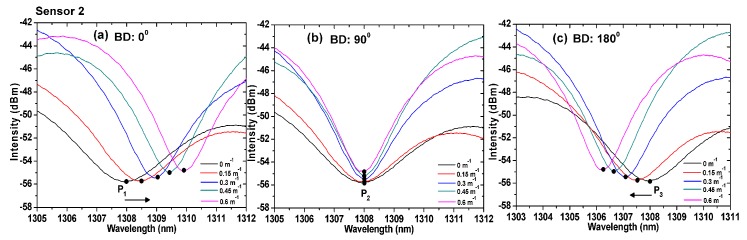
Spectra of TCF-based sensor 2 under different curvatures for bending directions of (**a**) 0°, (**b**) 90°, and (**c**) 180°. BD: Bending direction.

We performed a detailed analysis of the characteristics of the proposed sensors at different bending directions. Figure 6 and Figure 7 show the variations in the interference spectrum of the sensors for three different bending directions. The curvatures ranged from 0 to 1 m^−1^. At the 90° direction, the optical path difference between the core mode and cladding mode was constant because the cores were in the neutral plane. As a result, the interference minima D_2_ (1307.952 nm) and P_2_ (1308.024 nm) remained the same, as shown in Figure 6b and Figure 7b, respectively. However, the interference minima D_1_ and P_1_ at the 0° direction, shifted right (increasing wavelength) with the increase in the curvature, as shown in Figure 6a and Figure 7a, respectively. The interference minima D_3_ and P_3_ at the 180° direction, shifted left (decreasing wavelength) with the increase in the curvature, as shown in Figure 6c and Figure 7c, respectively.

**Figure 8:**
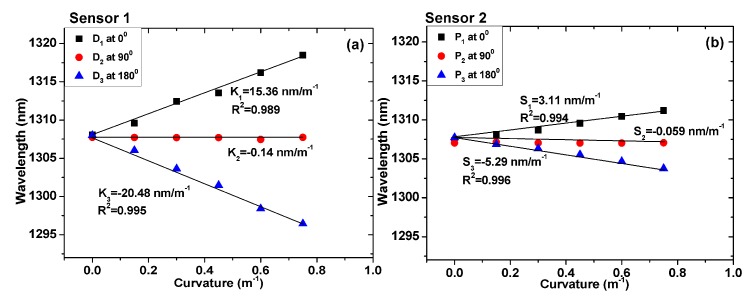
Wavelength shifting variation in the interference dip under different curvatures for bending directions of 0°, 90°, and 180°: (**a**) sensor 1 and (**b**) sensor 2.

To more clearly illustrate the bending phenomenon, Figure 8a,b show the wavelength shifting variation in the interference dip for the TCF-based sensors under different curvatures at bending directions of 0°, 90°, and 180°. It can be seen that the wavelength shifting variation in sensor 1 was larger than sensor 2, with the increase in curvature at the bending directions of 0° and 180°. This is because the eccentric core location of TCF in sensor 1 was further from the fiber’s central axis. However, the wavelength shifting variation of the two sensors remained the same at the bending direction of 90°. At the bending direction of 0°, the bending sensitivities of sensors 1 and 2 were 15.36 nm/m^−1^ and 3.11 nm/m^−1^, respectively. The correlation coefficients (R^2^) of the linear fitting were 0.989 and 0.994, respectively. In addition, the bending sensitivities of sensors 1 and 2 were −20.48 nm/m^−1^ and −5.29 nm/m^−1^, at a bending direction of 180°, respectively. It should be noted that at the 90° direction, the bending sensitivities of sensors 1 and 2 should have been zero. However, the measured values were −0.14 nm/m^−1^ and −0.059 nm/m^−1^, respectively, owing to the error in the bending direction. The correlation coefficients (R^2^) were 0.995 and 0.996, respectively. The correlation coefficients indicate that the linearity of the sensors is satisfactory in the measurement range.

Notably, the axial strain variation caused by the 5 g mass was so small that the mass-induced strain was ignored, and the wavelength shift of the sensors was considered to be resulting only from the bending. It has been theoretically proven that the wavelength shift of the sensor in the bending process is related to *d* (the distance between the eccentric core and the neutral plane during bending) and the curvature difference (Δ*C*) according to Equations (3) and (5). Specifically, if Δ*C* is constant, a greater distance between the eccentric core and the central axis increases the wavelength shift and produces higher corresponding sensitivity. It is clear from Figure 8a,b that, for the same bending direction and curvature, the sensitivity of sensor 1 was higher than that of sensor 2. Quantitatively, at the bending direction of 0°, the sensitivity of sensor 1 was 15.36 nm/m^−1^, which is 4.9 times higher than that of sensor 2. At the bending direction of 180°, the sensitivity of sensor 1 was −20.48 nm/m^−1^, which is 3.9 times higher than that of sensor 2. The distance between the eccentric core and the central axis of sensor 1 was 29.74 µm, which is larger than that of sensor 2. The result is consistent with the relation of the wavelength difference variation calculated by Equations (3) and (5). The optical loss effect of the sensor was not eliminated, which led to errors between the theoretical analysis and the experimental data.

Bending sensors based on a Fabry-Perot interferometer (FPI) [29], a FBG written on the ECF-SMF connection joint (FBG based on ECF) [30], a photonic crystal fiber (PCF-based MZI) [28], and a three-core fiber (TCF-based MZI) are listed in Table 1. This method improves the sensitivity by comparing different types of bending, which can be observed in Table 1.

### 3.3. Wavelength Shift of the TCF-Based Sensor in Temperature Experiments

**Figure 9:**
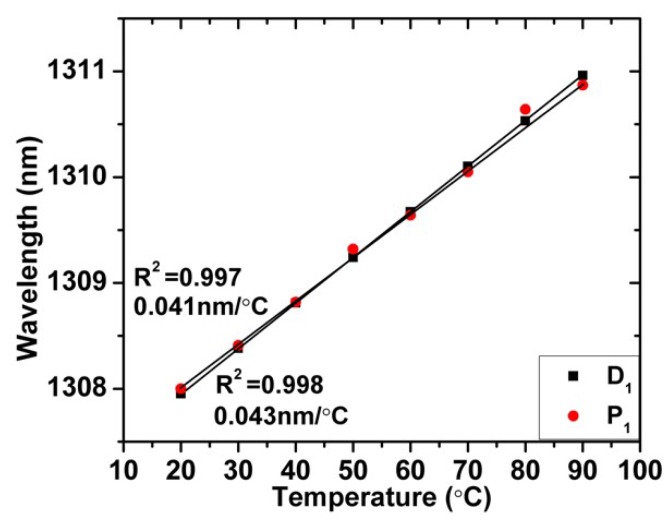
Wavelengths of interference minima D_1_ and P_1_ versus temperature.

The TCF-based sensors (sensors 1 and 2) were placed straight into the temperature-controlled chamber to measure their temperature characteristics from 20 to 90 °C. During the measurement, the polarization was kept constant by using the PC. To illustrate the temperature characteristics more clearly, Figure 9 shows the wavelength shifting of the interference minima against temperature. The temperature sensitivities of the interference minima D_1_ and P_1_ were 0.043 nm/°C and 0.041 nm/°C, respectively. The correlation coefficients (R^2^) were 0.998 and 0.997, respectively.

### 3.4. Limitations of the Research

The use of a TCF-based MZI sensor increased optical losses. During the experiments, a micrometer screw was pressed against the steel sheet to apply bending to the sensing head. Actual bending is more complex than the experimental simulation because different bending directions can be applied to the different parts of the sensing head simultaneously. In addition, care is required during sensor fabrication for the cleaving and fusion splicing procedures as the bare fiber sensor is fragile and can be easily broken.

### 3.5. Future Work

In the future, our research will focus on realizing miniaturized packaging for the TCF-based MZI sensor and applying the proposed sensors to biomedical sensing equipment. The repeatability of the sensor and reducing the loss of the optical system should be considered. A portion of the optical loss of the system could potentially be reduced by improved fabrication techniques.

## 4. Conclusions

In this study, two types of TCF-based MZI sensors were developed, and their sensing characteristics were investigated. The sensors had a short sensing element that makes it suitable for biomedical sensing. The experiments demonstrated the sensitive bending ability of the sensing structure by fusion-splicing the cores in the TCF at different locations between the two SMFs. The larger the distance between the eccentric core and the central axis, the greater the corresponding sensitivity. The bending sensitivities of the proposed sensors reached 15.36 nm/m^−1^ and 3.11 nm/m^−1^ at the bending direction of 0°, respectively. At the bending direction of 180°, the bending sensitivities of the sensors were −20.48 nm/m^−1^ and −5.29 nm/m^−1^. The temperature sensitivities of the two sensors were 0.043 nm/°C and 0.041 nm/°C, which had minimal effect on the experiments. The sensors are compact, versatile, inexpensive to fabricate, and are expected to have potential applications in biomedical sensing. 

## Figures and Tables

**Table 1:** Comparison of different types of bending sensors.

Subjects	Approach	Bending Sensitivities	
		0°	180°
1	FPI	242.5 pm/m^−1^	−231.5 pm/m^−1^
2	FBG based on ECF	49.3 pm/m^−1^	−50.3 pm/m^−1^
3	PCF-based MZI	5.129 nm/m^−1^ (No direction)	5.129 nm/m^−1^ (No direction)
4	TCF-based MZI	15.36 nm/m^−1^	−20.48 nm/m^−1^
